# Application of ITC-Based Characterization of Thermodynamic and Kinetic Association of Ligands With Proteins in Drug Design

**DOI:** 10.3389/fphar.2018.01133

**Published:** 2018-10-11

**Authors:** Haixia Su, Yechun Xu

**Affiliations:** ^1^CAS Key Laboratory of Receptor Research, Drug Discovery and Design Center, Shanghai Institute of Materia Medica, Chinese Academy of Sciences, Shanghai, China; ^2^School of Pharmacy, University of Chinese Academy of Sciences, Beijing, China

**Keywords:** ITC, thermodynamics, kinetics, protein–ligand recognition, drug design

## Abstract

A comprehensive characterization of the thermodynamic and kinetic profiling of ligands binding to a given target protein is crucial for the hit selection as well as the hit-to-lead-to-drug evolution. Isothermal titration calorimetry (ITC), widely known as an invaluable tool to measure the thermodynamic data, has recently found its way to determine the binding kinetics too. The extensive application of ITC in measurement of both thermodynamic and kinetic data manifests unique roles of ITC in drug discovery and development. This mini-review concentrates on elaborating how to gain the thermodynamic and kinetic data using ITC, highlighting the importance of these data in lead discovery and optimization, and intends to provide an overview of the technical and conceptual advances that offer unprecedented access to protein–ligand recognition by ITC measurement.

## Introduction

Thermodynamic and kinetic profiles of ligand–protein association have reawakened interest in drug discovery since they provide mechanistic insights into the molecular interactions determining the affinity of a ligand to its target and are useful to guide the compound (hit or lead) selection as well as the subsequent potency enhancement in the hit-to-lead-to-drug optimization (Olsson et al., 2008; Wang et al., 2017; Linkuviene et al., 2018). A thermodynamic characterization can provide more detailed information beyond the most important binding affinity, such as the driving force (enthalpic or/and entropic contribution) for the ligand association with its target. More and more examples manifested that thermodynamic parameters, in particular together with the hit–target complex structure, are indispensable for medicinal chemists to comprehensively understand the structure–activity relationship (SAR) and to gain useful information for hit selection or hit-to-lead optimization (Rechlin et al., 2017). Besides the thermodynamic parameters, the kinetic parameters of ligand–protein recognition shed light on the time-dependent changes of the binding event and are crucial for determining whether the compound is worth further development (Gaspari et al., 2016). A study has shown that the efficacy of the approved drugs has some relationship with the kinetic profiling of the drug–target binding, especially relevant to the residence time (Cramer et al., 2017a; Tonge, 2018). It is not difficult to understand that longer residence time would allow the target remain the affected state even when the concentration of the drug has decreased. This is also the major advantage of the drugs covalently binding to their targets as they have very long or even permanent residence time (Swinney, 2004; Zhang and Monsma, 2010).

Over the past three decades, isothermal titration calorimetry (ITC) has been accepted universally as a reliable tool to determining the thermodynamic parameters of inter-molecular interactions without any labeling (Ladbury and Chowdhry, 1996). A single titration experiment can gain the change of enthalpy (Δ*H*), the binding constant (*K*_a_), and the stoichiometry (n) of the reaction directly by analyzing the resultant titration curve. The Gibbs free energy (Δ*G*) and the change in entropy (Δ*S*) of the reaction can be calculated, according to the equation Δ*G* = Δ*H* − *T*Δ*S*. Most importantly, the hit–target binding affinity measurement by ITC can perfectly rule out the pan-assay interference compounds, referred to as PAINS, because ITC measures directly the heat flow of the hit–target binding rather than the change of fluorescence in many assays. Recently, based on the ITC instrument, a new method has been developed to measure the kinetic parameters of ligand–enzyme binding, expanding its use in drug discovery. A few examples about the application of ITC to determine kinetic parameters of ligand–enzyme binding have been successfully reported and a general protocol has been generated (Burnouf et al., 2012). In the ITC-based kinetic experiment, the power is plotted as a function of time and the parameters such as the association rate (*k*_on_) as well as the dissociation rate (*k*_off_) can be obtained directly and accurately according to the curve. As a result, for the purpose of identifying a promising hit with a slow dissociation rate, ITC supplies a powerful approach to evaluating the kinetic profiling of the hit binding to its target, no matter it is a non-covalent or covalent binder. Altogether, the advantage of ITC in measurement of both thermodynamic and kinetic parameters of the ligand–protein binding has made it a popular instrument in the field of medicinal chemistry.

Briefly, ITC is an invaluable tool in drug discovery not only because of its ability to measure the thermodynamic parameters with a single experiment, but also for its prospects for determination of the kinetic profiling of ligand–target binding. In the following, this mini-review will introduce how to apply ITC measurement to reveal the thermodynamic and kinetic signature of ligand–target binding, providing important information for hit selection and hit-to-lead optimization.

## Thermodynamic Characterization of Ligand–Target Binding

Although the commercially available ITC instrument has allowed more and more laboratories have access to measure the calorimetric data of ligand–protein binding, how to take full advantage of these data in the process of drug discovery remains to be explored. It is often the case that the *K*_d_ value derived from an ITC measurement is the only parameter with a certain reference value for hit/lead discovery while others such as Δ*H* and Δ*S* are ignored. Furthermore, it was once challenged whether the thermodynamic data should be taken into consideration in lead discovery (Geschwindner et al., 2015). On the other hand, studies have shown that compounds with better thermodynamic properties are more likely to become the best-in-class drugs (Freire, 2008). Besides, there have been lots of examples which successfully apply thermodynamic data to lead or drug discovery (Krimmer et al., 2016).

### Δ*H*, an Indicator for Hit-to-Lead Optimization

In regard to utilizing the ITC-measured thermodynamic signatures of ligand–protein binding to lead discovery, Klebe and his colleagues did the pioneering work and contribute enormously to this field (Krimmer and Klebe, 2015; Ruhmann et al., 2015). In their perspectives, even if the thermodynamic data do not have a good correlation with the potency of the hit, it is a key descriptor of the binding mode of a ligand and can be taken as an indicator for hit selection and optimization (Ghai et al., 2012). According to the equation, Δ*G* = Δ*H* – *T*Δ*S*, changes in enthalpy and entropy account for the Gibbs free energy of ligand binding. It is preferred to improve the enthalpy and the entropy simultaneously, but it is hard to be achieved because the gain in enthalpy is always accompanied with the loss of entropy. This is the pronounced enthalpy–entropy compensation, a phenomenon frequently observed in ligand–protein binding. It has been suggested that attention should be paid to the enthalpic contribution in the early state of lead discovery. A hit with an enthalpically more favorable binding signature is worthy of further development and hit-to-lead optimization should also seek to improve the binding enthalpy with a great effort (Ladbury et al., 2010). One reason for this is that it is easy to improve the entropic contribution but hard to improve the enthalpic one in the late state of lead discovery. As the lead discovery program proceeded, lipophilic groups are often added to the active ligand to improve its druggability, and synchronously the entropic property of the hit could be optimized due to the reduced flexibility. When the conformation of the ligand becomes more rigid, Δ*S* becomes smaller, indicating that the entropic loss of ligand-target binding is getting less. However, there is limitation for such an improvement resulted from the lipophilicity increase so that the enthalpic enhancement should be considered in advance. The other reason is that the more negative value of Δ*H* means more potent interactions formed between the ligand and its target, which has a significant impact on the selectivity improvement of a hit. In order to evaluate which ligand is more enthalpy favorable, enthalpic efficiency (EE = Δ*H*/Q, Q is the number of heavy atoms or molecular mass of a ligand) is proposed to rank ligands for hit selection and optimization. In line with this, a study shows that the best-in-class drug is generally more enthalpy favorable than the first-in-class drug by analyzing the thermodynamic profiling of inhibitors of HIV protease and the hydroxy methylglutaryl coenzyme A (HMG-CoA) reductase (Freire, 2008). In conclusion, Δ*H* serves as a key indicator during hit-to-lead optimization to qualify ligands as candidates for further modifications (Klebe, 2015).

### ITC Combined With Ligand–Protein Complex Structures

As mentioned above, the enthalpic efficiency is useful in selecting and optimizing the hit in the early state of lead discovery. Improvement of the enthalpic efficiency of a compound is the goal of optimization, but it is infeasible to carry out modification of the compound just based on the thermodynamic data. This might be the important issue leading many medicinal chemists to doubt whether the application of ITC in lead discovery is helpful. The rapid development of structure biology, in particular the X-ray protein crystallography, offers a powerful approach to efficiently solving the protein-ligand complex structures and revealing the actual binding mode of compounds in the binding pocket of targeting proteins, establishing the fundamental relationship of the thermodynamic data with protein–ligand interactions. Changes of protein–ligand interactions such as hydrophobic and hydrogen bonding (H-bonding) interactions are responsible for the enthalpy and/or entropy gain or loss. For example, introduction of a H-bond may increase or decrease in the ligand binding affinity, depending whether the enthalpy gains resulting from the newly formed H-bond is sufficient or not to compensate the enthalpy loss resulting from the disruption of previous H-bond(s) between water molecules and ligand/protein (Neeb et al., 2016). Therefore, only combining the complex structures with the thermodynamic data, can we understand the underlying mechanism of affinity changes resulting from the variation of protein–ligand interactions and establish a rational and validated SAR. Together with the protein–ligand complex structures, the thermodynamic profiling of ligand binding not only serves as an important indicator for hit or lead selection, but also interprets the nature of protein–ligand recognition and provides invaluable information for further optimization (Ruhmann et al., 2016).

The application of thermodynamic data in combination with complex structures in hit-to-lead optimization has been reported in several cases (Abel et al., 2008; Neeb et al., 2014; Cramer et al., 2017b). We also successfully utilized this strategy in the process of lead discovery of phosphodiesterase type 5 (PDE5) and fatty-acid binding protein 4 (FABP4), and found that the water molecule or water network in the ligand binding pocket plays an important role in association of compounds with targeting proteins. Herein, we delineate these two examples to demonstrate the power and advantage of this strategy in lead discovery.

**FIGURE 1:**
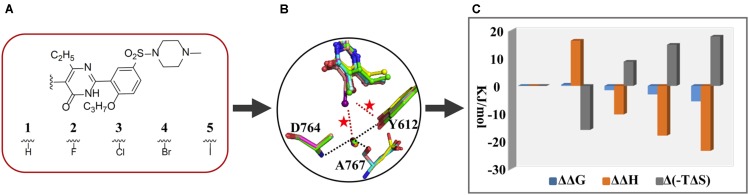
Application of thermodynamic data combined with complex structures in lead discovery of PDE5. **(A)** Chemical structures of five inhibitors. **(B)** Superimposition of inhibitors 1 (green), 2 (cyan), 3 (magenta), 4(yellow), and 5 (salmon), residues Y612, A767, and D764, and a structural water molecule by fitting the proteins of five crystal structures. **(C)** ΔΔ*G*, ΔΔ*H*, and Δ*(–T*Δ*S)* between one and the other four inhibitors.

Phosphodiesterase type 5, an enzyme catalyzing the hydrolysis of cGMP to 5-GMP, is an important drug target for treatment of diseases association with a low level of cGMP such as the pulmonary arterial hypertension and the erectile dysfunction (Giordano et al., 2001). Once our colleagues have identified the monocyclic pyrimidinones as novel and potent inhibitors of PDE5 (Wang et al., 2012; Gong et al., 2013). With this scaffold, a study manifesting the proof-of-concept of halogen bonding in drug design was carried out. Based on an initial docking conformation, a halogen atom (F, Cl, Br, and I) was used to replace the 5-position substituent of the pyrimidinone ring so as to introduce a halogen bond between the halogenated compounds and the residue Y612 of PDE5 (**Figure 1**). The determined crystal structures of PDE5 in complex with compounds 1 (H), 2 (F), 3 (Cl), 4 (Br), and 5 (I) revealed a similar binding mode of five compounds with the enzyme and the existence of the halogen bond between compounds 3/4/5 and Y612 (Xu et al., 2011). Subsequently, ITC experiments were performed to measure the strength of halogen bonding between compounds and PDE5 in order to provide the thermodynamic properties of halogen bonding in protein–ligand interactions for the first time (Ren et al., 2014). The revealed binding free energy of compounds 3, 4, and 5 (−34.9, −36.4, and −38.9 kJ/mol, respectively), gradually increased owing to a predominant enthalpic contribution (−41.4, −49.0, and −54.5 kJ/mol, respectively), which is attributable to the introduction of the halogen bond. This also means that the enthalpy gains completely compensate for the loss in entropic term resulting from the immobilization of Y612 and the halogenated inhibitors, overall increasing the binding affinity. It is thus revealed that the halogen bonding between the halogenated inhibitor and PDE5 is enthalpy driven. Compared to compound 1 in which the 5-H substituent has no interaction with PDE5, the ΔΔ*G* of 5-Cl, 5-Br, and 5-I binding to PDE5 is about −1.57, −3.09, and −5.59 kJ/mol, respectively, which is equivalent to the strength of the corresponding halogen bond.

In addition, the crystal structures show that the distances between OH of Y612 and the 5-Cl, 5-Br, and 5-I are 0.36, 0.34, and 0.37 nm, respectively, while the angle of C-Cl⋯OH, C-Br⋯OH, and C-I⋯OH is 141°, 149°, and 122°, respectively. Given these geometry data, it is reasonable to accept that a halogen bond is formed between the 5-Cl or 5-Br and OH of Y612, but it is not the case for the 5-I substituent. The paradoxically high binding strength in regard to the unfavorable bond length and angle inspired us to a closer inspection of the crystal structures, resulting the observation of a conserved water molecule which is simultaneously H-bonding to two residues and halogen bonding to the 5-I, 5-Br, or 5-Cl substituent. A following quantum mechanics calculation suggested that the interaction energy between the 5-Cl or 5-Br and Y612 is stronger than the energy between it and the water molecule, while it is just opposite in the case of the 5-I substituent. Therefore, the combination of thermodynamics with the X-ray crystallographic data, and the theoretical calculation not only present evidence for incorporation of the halogen bonding in protein–ligand binding affinity but also establish the first structure-energy relationship of halogen bonding. In particular, the discovery and characterization of the halogen bonding between iodine and the conserved water molecule provide a representative case with shedding light on the significance of halogen bonding to structural water for rational drug design.

**FIGURE 2:**
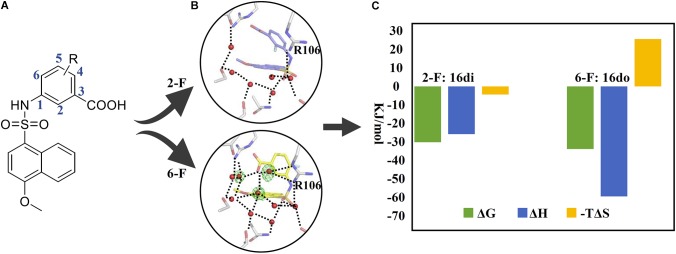
Application of thermodynamic data combined with complex structures in lead discovery of FABP4. **(A)** Chemical structure of inhibitors. **(B)** An important water network in the crystal structure of FABP4-16do (lower) and the disrupted water network in the crystal structure of FABP4-16di (upper). **(C)** The binding free energy (Δ*G*), enthalpy (Δ*H*), and entropy term (*–T*Δ*S*) of 16di and 16do binding to FABP4.

The second example which takes advantage of the thermodynamic data complemented by complex structures is concerning the lead discovery of FABP4. FABP4, mostly expressed in adipose cells, is a fatty-acid binding protein responsible for transportation of a series of saturated fatty acids to mediate cell signal pathways. Studies have shown that FABP4 knockout mice were protected from diseases such as atherosclerosis and the type 2 diabetes mellitus (T2DM; Furuhashi and Hotamisligil, 2008). We have discovered the naphthalene-1-sulfonamide derivatives as novel inhibitors of FABP4, in which congeneric compounds 16di and 16do possessing a fluorine atom at the C-2 and the C-6 position of the phenyl ring, respectively, were quite different in their activities against FABP4 (Gao et al., 2018; **Figure 2**). In order to provide an explanation for the SAR, we first solved the crystal structures of FABP4 bound with 16di and 16do, revealing that the binding modes of two compounds are quite similar except a minor difference in orientation of the fluorinated phenyl ring. Further inspection of the complex structures found that the water-molecule network in the FABP4-16di complex structure was disrupted because of the small shift of the phenyl ring, resulting the broken of the H-bonding interactions between the carboxyl oxygen of 16di and R106. To gain deep insight into how the network surrounding the compound together with the orientation change of the phenyl ring affects the binding affinity, the thermodynamic profiling of 16do and 16di binding to FABP4 were measured with ITC. The Δ*H* of 16do and 16di binding to FABP4 was −59.64 and −25.87 kJ/mol, respectively, suggesting that the binding of two compounds to FABP4 was enthalpy driven. However, when compared to 16di, the enthalpy gains of 16do are more significant due to the presence of the complete water-molecule network and stronger interactions with the surrounding residues as well as water molecules. In contrast, the binding of compound 16di harvested a more advantageous entropic term (−*T*Δ*S*: −25.72 kJ/mol) ascribed to the disruption of the water-molecule network compared to compound 16do (−*T*Δ*S*: −4.38 kJ/mol). Accordingly, the complex structures associated with thermodynamic data demonstrates that the water-molecule network in the binding pocket of FABP4 should be taken into consideration in the process of lead discovery.

Both examples presented above revealed the important role of water molecules in lead discovery and the interest should be reawakened in the pocket water molecules (Wienen-Schmidt et al., 2018). Although attention has been paid to the key water molecules (Betz et al., 2016) and some softwares such as WaterMap have been intendedly developed to explore the pocket water molecules (Young et al., 2007), the impact of the versatile and ubiquitously present water molecules is hardly understood and the involvement of water molecules is a major cause for the inherent complexity of protein-ligand recognition (Spyrakis et al., 2017). How to fully take the water molecules into account in drug design is challenging yet. Nevertheless, a surprising enhancement in binding affinity may come true if we take advantage of these mysterious water molecules based on the information provided by the thermodynamic profiling and complex structures.

Overall, a comprehensive analysis of the protein–ligand binding based on the complex structures complemented by the thermodynamic profiling is capable of providing mechanistic insights into the molecular interactions determining the affinity of a ligand to its target, in particular those of unexplored or ignored factors that contribute to the protein–ligand recognition. It is supposed to strengthen our understanding of the complex protein–ligand binding event and to offer the medicinal chemistry community useful tools for rational drug design.

## Kinetic Data Measurement

It is already accepted that ITC measurement can be used to obtain binding constants (*k*_m_, *k*_cat_, and *V*_max_) of enzyme–substrate reactions and inhibitory constant (*K*_i_) of the enzyme–inhibitor binding, and the employment of this in studies of different enzymes has gained a lot of interest (Todd and Gomez, 2001; Lehoczki et al., 2016). However, a putative use of ITC to measure the binding kinetics of ligands to its target remains unaccomplished until Di Trani et al. developed a new method, called a kinetic ITC technique, that can measure binding kinetics (*k*_on_ and *k*_off_) and the inhibitory constant (*K*_i_) of inhibitors simultaneously (Di Trani et al., 2018), expanding the application of ITC in characterization of the protein-ligand binding process. A simple competitive enzyme inhibition model described by the following kinetic scheme was used:

(1) $$\text{EI}\overset{k_{\text{off}}}{\underset{k_{\text{on}}}{⇄}}\text{E}+\text{I}+\text{S}\overset{k_{1}}{\underset{k_{-1}}{⇄}}\text{ES}\overset{k_{\text{cat}}}{\to }\text{E}+\text{P}$$

where E, S, P, I, and EI represent the enzyme, substrate, product, inhibitor, and enzyme-inhibitor complex, respectively. *k*_on_, *k*_off_, and *k*_cat_ are the association rate, the dissociation rate, and the catalytic rate, respectively. The instantaneous rate of enzyme catalysis is given by the Michaelis–Menten equation:

(2) $$\frac{\text{d}[\text{P}]}{\text{dt}}=-\frac{\text{d}[\text{S}]}{\text{dt}}=\frac{{\text{k}}_{\text{cat}}[\text{S}]([{\text{E}}_{0}]-[\text{EI}])}{[\text{S}]+{\text{k}}_{\text{m}}}$$

where [P], [S], [E_0_], and [EI] are the concentrations of product, substrate, total enzyme, and enzyme-inhibitor complex, respectively. *k*_m_ is the Michaelis constant. Inhibition was assumed to follow the first-order kinetics according to:

(3) $$\frac{\text{d}[\text{EI}]}{\text{dt}}=k_{\text{on}}[\text{E}][\text{I}]-k_{\text{off}}[\text{EI}]$$

where [E] is the concentration of free enzyme. The heat flow, Q(t), generated by catalysis, was calculated according to:

(4) $$\text{Q}(\text{t})=\Delta {\text{H}}_{\text{cat}}{\text{V}}_{\text{cell}}\frac{\text{d}[\text{P}]}{\text{dt}}$$

where ΔH_cat_ is the enthalpy of catalysis and V_cell_ is the volume of the cell, followed by convolution with the empirical instrument response function. The basis of this new method is that ITC detects heat flow in real time, and thus give a direct readout of enzyme activity and its variation in response to inhibitor binding according to Eqs 2 and 4. Then the kinetic parameters of inhibitor binding to enzyme can be calculated according to Eq 3.

In general, two kinds of experiments, named the kinetics of inhibition experiment and the kinetics of initiation experiment, are required to gain the kinetic property of inhibitor binding to enzyme. In the former one, the inhibitor in the syringe is titrated into the cell containing the enzyme and its substrate, and the resulting power values are supposed to shift upward. It is not hard to understand that the change of power is highly proportional to the amount of inhibited enzyme while the velocity of the change has something to do with the velocity of the inhibitor associating with the enzyme. Consequently, the value of *K*_i_ and *k*_on_ can be resulted directly from the kinetics of inhibition experiment. In the kinetics of initiation experiment, the enzyme already saturated by inhibitors in the syringe is titrated into the cell containing the substrate, and the power will shift downward. Accordingly, *K*_i_ and *k*_off_ can be determined with this experiment. This kinetic ITC technique is able to measure the kinetic property of inhibitors with *k*_on_ from 10^3^ to 10^7^ M, T from 10 s to 30 min, and *K*_i_ limited to sub-nM.

Di Trani et al. utilized this kinetic ITC technique to successfully determine the kinetic parameters of covalent and non-covalent inhibitors binding to the prolyl oligopeptidase (POP), a promising target for the treatment of cancer and neurodegenerative disorders (García-Horsman et al., 2007). They characterized binding kinetics of five compounds to POP using a Malvern ITC-200 calorimeter. Two of them bind non-covalently to POP, while another three form reversible covalent bonds with the catalytic serine in the POP active site via aldehyde or nitrile moieties. In order to further test the reliability, they used spectroscopic methods to validate the ITC kinetics measurements and found that the range of applicability of the ITC kinetics experiments is far greater due to the shorter delay between mixing and detection and the greater sensitivity to changes in catalytic rate.

Currently, the application of the kinetic data resulted from ITC measurement to lead discovery is rare, however, the prospects for engagement of ITC in kinetic data determination are bright as the ITC kinetic technique is prevalent, accurate, and time-saving as well. However, there are also some limitations of the kinetic ITC technique. For example, it is not applicable to measure the kinetics of non-enzyme perturbation since this technique has to detect the heat directly resulted from the enzyme reaction. Besides, the lack of a convenient suite to analyze data has hindered the wide application and extension of this technique.

## Conclusion

Isothermal titration calorimetry is becoming an indispensable tool with the ability of determining the thermodynamic as well as kinetic parameters associated with protein–ligand recognition, playing a pronounced role in drug design. Although the thermodynamic profiling alone can provide information for hit selection and hit-to-lead optimization, when combined with the high-resolution complex structures, it will give us sufficient knowledge on the inherent factors determining the binding affinity and will provide deep insight into how the unpredictable water molecules, in particular those in the pocket, influence the protein–ligand binding event. The examples regarding the lead discovery of PDE5 and FABP4 cast light on the power of application of thermodynamic data together with the crystal structures in drug design and in exploring the crucial roles of a water molecule or water network in binding of ligands to targets. An unexpected improvement in binding affinity may be interpreted and achieved if we take the pocket water into account, though efficient utilization of these key water molecules in rational drug design remains a challenge. In addition, ITC is promising to be a universal method for evaluation of the kinetic parameters of ligands binding to enzymes, which is important to find out the lead or candidate drug with a desirable residence time. In this respect, a mature commercial suite needs to be further developed in order to analyze the data automatically. Therefore, it is anticipated that continued efforts on the ITC-based characterization of the protein–ligand binding with a wealth of thermodynamic and kinetic data will lead to novel findings that will enhance our ability to understand the full spectrum of protein–ligand recognition and drug action, and appropriately capitalize on these findings for medical applications.

## Author Contributions

HS and YX wrote the manuscript.

## Conflict of Interest Statement

The authors declare that the research was conducted in the absence of any commercial or financial relationships that could be construed as a potential conflict of interest.
